# Mandibular Advancement Device Therapy in 182 DISE-Selected Adults with Moderate-to-Severe Obstructive Sleep Apnea: A Multicenter Real-World Study

**DOI:** 10.3390/biomedicines14071652

**Published:** 2026-07-22

**Authors:** Marc J. Braem, Muriel Lins, Annelies Van Den Bergh, Frans Verhelst, Leslee Willes, Ellen Collier

**Affiliations:** 1 Department of Translational Neurosciences, Faculty of Medicine and Health Sciences, University of Antwerp, 2610 Wilrijk, Belgium; 2 Department ENT, Oral Appliance Clinic, University Hospital Antwerp, 2650 Edegem, Belgium; xellencollier@yahoo.com; 3 Department of Pulmonology, AZ Sint-Maarten, 2800 Mechelen, Belgium; muriel.lins@emmaus.be; 4 Department of Pulmonology, Imelda Ziekenhuis, 2820 Bonheiden, Belgium; annelies.van.den.bergh@imelda.be; 5 Department of Pulmonology, Heilig Hart Lier, 2500 Lier, Belgium; frans.verhelst@telenet.be; 6 Willes Consulting Group, Inc., San Diego, CA 92024, USA; lesleew@willesconsulting.com; 7 Department of Maxillofacial Surgery, AZ Voorkempen, 2390 Malle, Belgium; 8 Department of Dental Sleep Medicine, Heilig Hart Lier, 2500 Lier, Belgium; 9 Department of Dental Sleep Medicine, AZ Monica, 2018 Antwerpen, Belgium

**Keywords:** mandibular advancement device, oral appliance therapy, obstructive sleep apnea, OSA, upper-airway collapse, ESS, snoring

## Abstract

**Background/Objectives**: The study evaluates the effectiveness of mandibular advancement device (MAD) treatment in drug-induced sleep endoscopy (DISE) preselected adults with moderate-to-severe obstructive sleep apnea (OSA). **Methods**: It is a retrospective observational cohort study without a control arm/treatment, including 182 patients with an apnea–hypopnea index (AHI) ≥ 15 events/h of sleep (Level-1 polysomnography) from six Belgian hospitals. After DISE preselection, a custom-made MAD (EVO, ProSomnus, Pleasanton, CA, USA) was titrated to symptom relief or physiological limits. Level-3 home polygraphy with MAD was performed within five months. The primary outcome was change in AHI from baseline. Secondary outcomes included percentage change in AHI, change in OSA severity category, snoring loudness and Epworth Sleepiness Scale (ESS) scores. Analysis of covariance methods adjusting for baseline AHI were employed. Exploratory analyses compared outcome measures by OSA severity at baseline. ‘Success’ equaled AHI < 10 with ≥50% improvement. **Results**: Mean AHI significantly decreased from 24.4 to 7.1 (*p* < 0.0001) with a significant mean percent decrease by 68.6% (*p* < 0.0001), improving more (*p* < 0.0001) in severe cases. Mean change in snoring loudness improved significantly from a mean of 7.1 to 1.7 (*p* < 0.0001), improving more (*p* = 0.049) in moderate cases. Mean change from baseline in overall ESS score was −1.7 (*p* < 0.0001). Greater improvements were observed in participants with elevated baseline ESS scores. MAD significantly (*p* = 0.03) improved OSA category: moderate OSA cases improved by a mean of 1.4 levels, severe cases by a mean of 2.1 levels (*p* < 0.0001). A total of 75.8% of participants achieved ‘Success’. **Conclusions**: In this retrospective multicenter cohort, MAD therapy was associated with significant reductions in AHI, snoring loudness, and ESS scores among DISE-preselected adults with moderate-to-severe OSA. Because treatment outcomes were assessed using Level-3 home polygraphy, the findings should be interpreted as real-world effectiveness outcomes obtained within the Belgian clinical care pathway.

## 1. Introduction

Obstructive sleep apnea (OSA) is a highly prevalent disorder [1], affecting an estimated 936 million adults worldwide aged 30 to 69 years with mild to severe disease. OSA is associated with increased morbidity and mortality, particularly through adverse cardiovascular consequences [2]. It is characterized by recurrent episodes of complete (apnea) or partial (hypopnea) upper-airway obstruction during sleep, resulting in intermittent oxygen desaturation, sleep fragmentation, and non-restorative sleep [3,4].

Currently, there are two non-invasive evidence-based therapies for the treatment of OSA aimed at widening and stabilizing the upper airway to preserve breathing [5]. The conventional therapy is continuous positive airway pressure (CPAP), creating upper airway patency by pneumatic splinting. The second is mandibular advancement device (MAD) therapy which exercises mechanical traction via mandibular protrusion to open and stabilize the upper airway. To improve patient selection, DISE has become increasingly standardized and is now widely used to evaluate upper-airway collapse patterns and predict treatment response [6].

Treatment effectiveness is commonly assessed using the apnea–hypopnea index (AHI) defined as the average number of apneas and hypopneas per hour of sleep [7]. Although MAD therapy is generally associated with smaller reductions in AHI than CPAP, it often achieves higher long-term adherence [5,8,9,10,11]. Consequently, MAD therapy remains an important treatment option for patients with OSA.

However, the existing MAD literature is characterized by substantial heterogeneity in study design, sample size, and device type [8,12]. Many studies include relatively small cohorts or combine multiple oral appliance designs, making it difficult to determine the effectiveness of individual devices [12,13]. Because MAD design influences therapeutic outcome [12,14,15], conclusions derived from one appliance cannot necessarily be generalized to another. Large, multicenter studies evaluating specific MAD designs under routine clinical conditions remain limited.

Randomized controlled trials are considered the gold standard for evaluating treatment efficacy but often include highly selected participants treated under controlled conditions. In contrast, real-world observational studies may better reflect routine clinical practice by including broader patient populations. Together, both approaches are needed to understand not only whether a treatment can work under ideal conditions, but also whether it works in everyday clinical care [16,17,18].

MAD therapy is currently recommended primarily for mild-to-moderate OSA and as an alternative treatment for patients who do not tolerate or respond to CPAP [19,20,21]. In Belgium, treatment effectiveness following MAD therapy is routinely evaluated using Level-3 home polygraphy (HPG) within the national reimbursement pathway [22,23]. Consequently, outcome assessment in routine clinical practice differs from the polysomnography-based follow-up commonly used in controlled research settings.

The objective of this retrospective multicenter study was to evaluate the real-world effectiveness of a specific MAD design in 182 DISE-selected adults with moderate-to-severe OSA treated across six general hospitals. Treatment effectiveness was assessed using changes in AHI, snoring loudness, and daytime sleepiness over a six-month observation period. Outcomes were evaluated overall and according to baseline OSA severity. Because follow-up was performed using Level-3 HPG, the study was designed to assess real-world treatment effectiveness rather than efficacy under controlled polysomnographic conditions.

## 2. Materials and Methods

### 2.1. Clinical Pathway

Patients suspected of OSA underwent diagnostic evaluation according to the Belgian clinical care pathway, including attended Level-1 polysomnography (PSG) for diagnosis [22,23,24].

For the present retrospective cohort study, diagnostic records were obtained from six Belgian sleep centers: AZ Monica (AZMO, Antwerpen, Belgium), AZ Sint-Maarten (AZSM, Mechelen, Belgium), AZ Voorkempen (AZVK, Malle, Belgium), Heilig Hart Ziekenhuis (HHAR, Lier, Belgium), Imelda Ziekenhuis (IMEL, Bonheiden, Belgium), and VITAZ (VITA, Sint-Niklaas, Belgium). Sleep study details are provided in Appendix A.1. All PSG recordings were scored in 30 s epochs according to AASM 2018 criteria by qualified sleep technicians. There was no calibration of the sleep studies over the participating centers.

Patients with moderate-to-severe OSA (AHI ≥ 15 events/h) considered for MAD therapy were referred to an ear, nose and throat (ENT) specialist experienced in sleep medicine for awake upper-airway evaluation [25]. Subsequently, a dental examination was performed by the affiliated dental sleep medicine team. Mandibular protrusion was recorded using a bite registration protocol as described below.

During the drug-induced sleep endoscopy (DISE) procedure, mandibular protrusion was simulated using the previously obtained bite registration. A positive DISE response was defined as a clinically relevant improvement in upper-airway patency following mandibular advancement, as judged by the experienced ENT specialist. Patients demonstrating complete concentric collapse at the level of the soft palate without improvement during mandibular protrusion were generally not considered suitable candidates for MAD therapy. Because this retrospective multicenter study reflected routine clinical practice across six hospitals, no mandatory standardized DISE scoring system (e.g., VOTE classification) or formal inter-center calibration was imposed. DISE findings were therefore interpreted by experienced ENT specialists according to their training and local clinical practice [26].

Referral for MAD therapy followed discussion of treatment options between the treating physician and the patient.

At the time of device fitting, participants received instructions regarding titration and were advised to use the MAD for at least four hours per night. Device use was self-reported [27].

Treatment effectiveness was assessed using Level-3 HPG as detailed in Appendix A.2. Because HPG uses total recording time rather than total sleep time, respiratory events were scored relative to recording time. Scoring followed current AASM recommendations for cardiorespiratory monitoring.

Questionnaires were administered at baseline, one month, and at the end of the observation period. Snoring severity was assessed using a visual analog scale for snoring (VASS), ranging from 0 (no snoring) to 10 (partner sleeps in another room). Daytime sleepiness was assessed using the Epworth Sleepiness Scale (ESS), ranging from 0 to 24.

Because the study was based on routinely collected clinical data, transient adverse effects associated with adaptation to MAD therapy (e.g., jaw discomfort, oral dryness, hypersalivation, or minor occlusal changes) were not systematically recorded. However, the ability of participants to initiate treatment at the prescribed starting position (MCP-2) and any documented treatment modifications due to intolerance were collected.

### 2.2. Intervention

**Figure 1:**
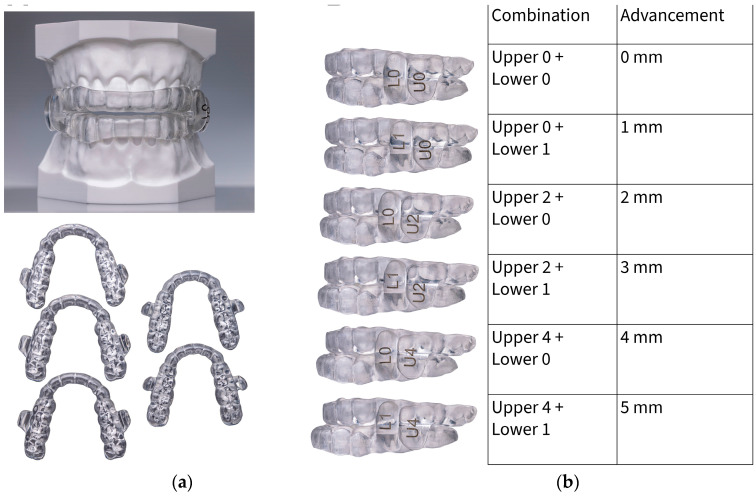
Mandibular advancement device used and titration schedule. (**a**) Illustrates the perpendicular posts maintaining mandibular protrusion; (**b**) shows that each post has a mark indicating upper (U) or lower arch (L) followed by a number: “0” or “1” for the lower arch, and “0”, “2” or “4” for the upper arches (right). The sum of the digits for upper plus lower arches yields the applied amount of protrusion. In the present study, the start of treatment position (maximal comfortable protrusion minus 2 mm) equals to U0/L0. If additional mandibular advancements are needed, titration is achieved by moving up to the next larger appliance number, in 1 mm increments.

The MAD used in this study (EVO, ProSomnus Sleep Technologies, Pleasanton, CA, USA) (Figure 1) was manufactured from a solid block of Class VI (MG6) medical-grade resin without incorporating premanufactured components [28]. The design incorporates perpendicular rectangular posts that maintain mandibular advancement [14] also in the presence of limited mouth opening during sleep.

At the time of bite registration, the full mandibular protrusive range was recorded (the BiteFix, Scheu-Dental, Iserlohn, Germany) with a hard bite registration paste (Futar Fast, Kettenbach, Germany). Also, the ‘maximal comfortable protrusion’ (MCP, mm) was determined, being the most forward mandibular position still tolerated by the patient.

The starting position of the MAD therapy was then set at MCP minus 2 mm (MCP-2, mm). Digital imprints of both tooth arches (Trios 3 wireless pod, 3Shape, Copenhagen, Denmark) and of the MCP-2 position were taken. The fully digital patient records guided the individual manufacturing process.

Participants were instructed to titrate the MAD (Figure 1) until resolution of snoring symptoms and/or daytime sleepiness, or until physiological limits were reached.

### 2.3. Participants

Consecutive patients treated in routine clinical practice at six independent hospitals between March 2023 and June 2024 were included. Screening data were collected at study entry. Treatment initiation occurred according to routine clinical scheduling at each participating center. End dates were selected following the governmental timeline which mandates the confirmation of treatment effectiveness within five months of treatment initiation during a six-month follow-up period.

The inclusion criteria were consistent with standard clinical and health governmental indications, without the application of highly selective eligibility requirements, reflecting real-world clinical practice. To be eligible for the study, patients were required to have an AHI ≥ 15 events/h diagnosed with a Level-1 PSG not older than two years at the time of enrolment. They also had to be dental fit and demonstrate the absence of complete circular collapse at the palate with the bite registration during DISE.

A total of 182 patients were included in the study. Baseline characteristics of the study population were collected.

All participants provided written consent for their routine clinical treatment prior to initiation of MAD therapy. The study “Real-World Assessment of Clinical Evidence for Mandibular Advancement Treatment of Obstructive Sleep Apnea (RACEMADT)” was registered at www.clinicaltrials.gov with identifier NCT06837285.

### 2.4. Variables

The baseline AHI (events/h) was derived from the PSG diagnosis and body mass index (BMI; kg/m^2^) was calculated. Oxygen desaturation metrics were not included in the predefined analyses.

Baseline characteristics including age (years) and sex (Female/Male, F/M) were collected. VASS score and ESS score were completed at baseline, 1-month follow-up session and at the end of the 6-month observation period. A VASS score > 3 (out of 10) was considered indicative of socially disturbing snoring, while an ESS score ≥ 11 was considered indicative of excessive daytime sleepiness. The change from baseline was evaluated for AHI, VASS and ESS parameters, thereby each patient serving as their own control.

The primary functional objective outcome measure was the change in AHI from baseline. Secondary functional objective measures include summarizing AHI as the percent change from baseline and the change in OSA severity category. Additionally, ‘Success’ was defined as treatment AHI < 10 events/h and at least 50% improvement in AHI from baseline. The secondary functional subjective measures are the change in VASS score and ESS score from baseline.

Changes from baseline in all outcome measures were evaluated overall and compared between baseline OSA severity categories.

### 2.5. Statistical Methods

The sample size of 182 participants was considered sufficient to estimate the overall change in AHI with a precision of 1.5 events/h (95% margin of error) assuming a standard deviation of 10 events/h.

Data were analyzed with SAS^®^ Version 9.4 (SAS Institute Inc., 100 SAS Campus Drive, Cary, NC, USA). Significance level was set at α = 0.05 using two-sided statistical tests.

Outcome measures consisted of continuous variables (AHI), categorical variables derived from AHI (OSA severity category, treatment success), or score variables (VASS score, ESS score). Descriptive statistics include the number of observations, mean, standard deviation (or standard error) and median for continuous parameters, and frequency counts and percentages for categorical parameters. Ninety-five percent confidence intervals were calculated for estimating changes from baseline.

Baseline characteristics (age, sex, BMI, AHI, VASS score, ESS score, OSA severity) were compared across centers using Fisher’s exact tests for categorical variables and analysis of variance for continuous variables to confirm balanced characteristics across centers. Poolability was assessed using change in AHI from baseline. Initial univariate analyses evaluated center effects alone, followed by multivariate analyses adjusting for baseline AHI and other unbalanced baseline characteristics.

Overall mean change from baseline in functional objective and subjective outcome measures after MAD (change in AHI, percent change in AHI from baseline, change in OSA categories, change in VASS score and change in ESS score) were analyzed using analysis of covariance methods adjusting for baseline AHI. Changes in functional objective and subjective outcome measures from baseline were also stratified across baseline OSA severity categories (moderate vs. severe) and compared between categories using a Student’s *t*-test. A subset of participants considered ‘sleepy’ with baseline ESS score ≥ 11 (out of 24) was summarized in the same fashion as overall ESS-scores.

Treatment success was reported overall and compared between baseline OSA severity categories using Fisher’s exact test. A trend test comparison of OSA severity categories from baseline to follow-up was tested with Cochran–Mantel–Haenszel Statistics based on Table Scores.

The analysis focused retrospectively on treated participants, providing insights into care patterns, adherence, and outcomes in routine practice. All participants completed the 6-month observation period including follow-up HPG. No data were missing for any of the study variables, so no imputation or handling of missing data was necessary, and all analyses were performed on the complete dataset.

## 3. Results

All 182 selected participants were confirmed eligible and completed the full governmental care pathway from diagnosis through DISE to MAD treatment with follow-up.

The baseline characteristics of the study population are summarized in Table 1 and Table 2. Participants were predominantly middle-aged, male, and overweight. The cohort consisted exclusively of participants with moderate-to-severe OSA, with moderate OSA representing the largest subgroup. Mean baseline ESS scores were below the threshold for excessive daytime sleepiness in the overall cohort. Participants in the subgroup exhibited elevated excessive daytime sleepiness.

In the present study, therapy outcome confirmation with Level-3 HPG was done after a median of 85.0 (69.0–107.3) days and the median observation period lasted 126.0 (102.0–148.3) days.

### 3.1. Baseline Characteristics and Poolability

Significant differences in baseline characteristics across centers as presented in Table 1 and Table 2 were noted for AHI (*p* = 0.01), age (*p* = 0.0004), sex (*p* = 0.002), BMI (*p* = 0.03), and ESS score (*p* = 0.01), but not for VASS score or OSA severity category.

**Figure A1:**
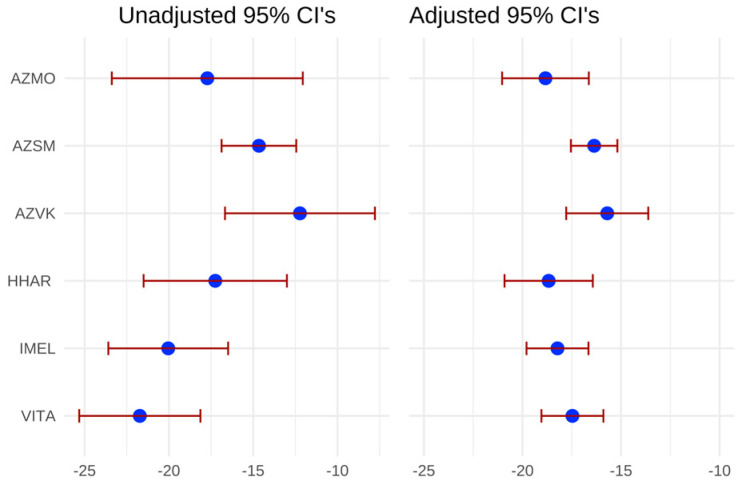
Unadjusted and adjusted confidence intervals for the mean changes in AHI from baseline according to the participating centers: AZMO (AZ Monica); AZSM (AZ Sint-Maarten); AZVK (AZ Voorkempen); HHAR (Heilig Hart Ziekenhuis Lier); IMEL (Imelda Ziekenhuis Bonheiden); VITA (VITAZ); AHI (apnea–hypopnea index, events/h sleep); CI’s (95% confidence intervals for the mean).

**Figure A2:**
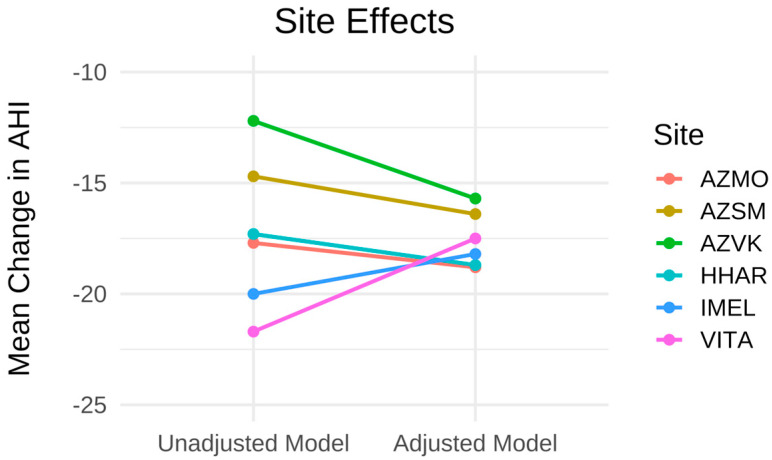
Estimated Site Effects in the Unadjusted and Adjusted Models. Unadjusted model includes center only as an explanatory variable; Adjusted model additionally includes covariates of baseline AHI (apnea-hypopnea index), baseline BMI (body mass index) and age. AZMO (AZ Monica); AZSM (AZ Sint-Maarten); AZVK (AZ Voorkempen); HHAR (Heilig Hart Ziekenhuis Lier); IMEL (Imelda Ziekenhuis Bonheiden); VITA (VITAZ).

Poolability was tested across centers using the primary functional outcome measure, change from baseline in AHI (Table 3). Based on a univariate approach considering center as the only explanatory variable, the data were not poolable (*p* = 0.002). An additional poolability analysis was generated using a multivariate approach, including baseline AHI and unbalanced baseline characteristics as explanatory variables in the model. The effect of center on change in AHI became non-significant (*p* = 0.10) when accounting for baseline AHI and other unbalanced baseline characteristics. As evident in Figure A1 and Figure A2 in Appendix B the adjusted 95% confidence intervals are more comparable between centers after adjusting for baseline characteristics. The lack of poolability observed in the univariate analysis was attributable to differences in baseline characteristics across the participating Belgian general hospitals rather than treatment outcome. Consequently, subsequent analyses were conducted on pooled data adjusted for baseline AHI and focused on the care pathway in Belgium.

### 3.2. Functional Objective Outcomes

**Figure 2:**
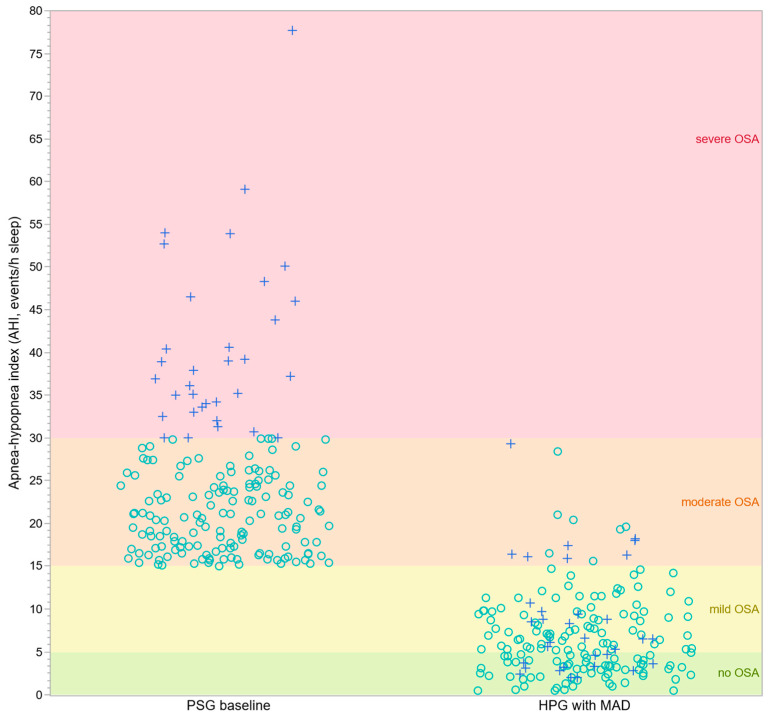
Effect of mandibular advancement device treatment on AHI, measured using different sleep-study levels: the AHI at baseline was derived from PSG and upon mandibular advancement device treatment from HPG. Individual datapoints are grouped according to moderate (o) and severe (+) OSA severity. The background colors indicate OSA categories in green (no OSA), yellow (mild OSA), orange (moderate OSA) and red (severe OSA).

**Figure 3:**
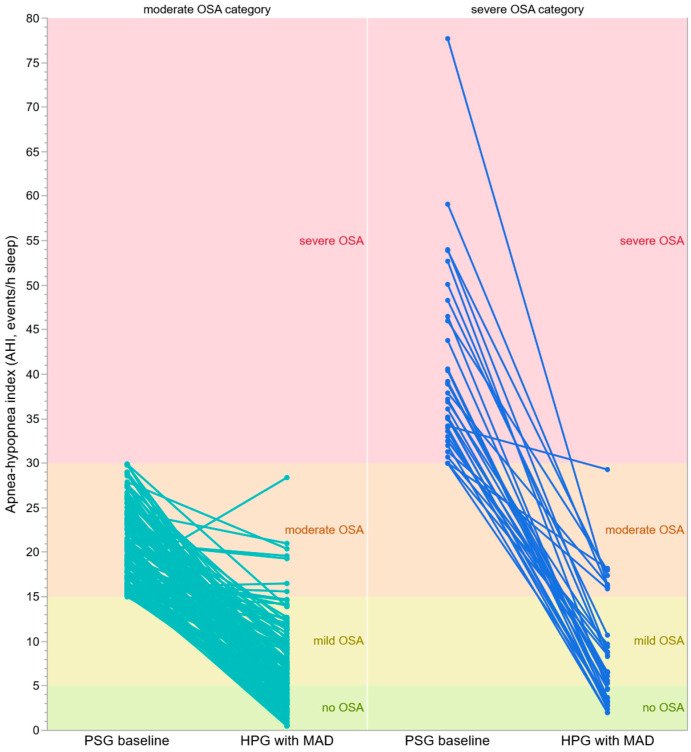
Pairwise presentation of AHI from baseline (measured with Level-1 PSG (**left**)) to follow-up with mandibular advancement device (measured with Level-3 HPG (**right**)) according to OSA category. The background colors indicate OSA categories in green (no OSA), yellow (mild OSA), orange (moderate OSA) and red (severe OSA).

Figure 2 and Figure 3 illustrate AHI values obtained using different sleep-study modalities: baseline AHI was derived from Level-1 PSG and follow-up AHI during MAD therapy from Level-3 HPG. Figure 2 presents the individual AHI measuring points at baseline and following MAD therapy overlaid on the corresponding OSA severity category.

Figure 3 presents the paired AHI values at baseline and upon mandibular advancement device therapy overlaid on the corresponding OSA severity category.

Table 4 presents mean AHI at baseline and treatment follow-up and the associated mean change from baseline and percent change from baseline. There was a significant reduction in the mean change in AHI of −17.3 ([95% CI, −18.0 to −16.6]; *p* < 0.0001) events/h compared to baseline. The mean percent change from baseline in AHI also demonstrated a significant reduction of −68.6% [95% CI, −72.0% to −65.2%); *p* < 0.0001]. A sensitivity analysis of mean change in AHI was conducted using a mixed-effects model with center as a random effect as additional confirmation of poolability across centers. The results were similar to those reported above, with mean change in AHI of −17.3 ([95% CI, −18.2 to −16.5]; *p* < 0.0001) events/h compared to baseline.

MAD therapy resulted in a mean AHI of 6.7 (SD 4.7) events/h for moderate OSA participants and 8.7 (SD 6.4) events/h for severe participants. The mean change in AHI as well as the percent change in AHI from baseline was significantly different for participants with severe OSA compared to those with moderate OSA (*p* < 0.0001 and *p* = 0.003, respectively) as shown in Table 5.

To identify factors associated with treatment response, multivariate regression analyses were performed including baseline AHI, age, BMI, baseline VASS score, and baseline ESS score. Baseline AHI was the strongest predictor of change in AHI with an effect size of −0.88, followed by age with an effect size of 0.18 and BMI with an effect size of 0.10. Sex, baseline VASS score, and baseline ESS score were not significant predictors. No statistically significant pairwise interactions were observed.

A significant reduction in the OSA category from baseline to the end of the observation period for all participants combined (*p* = 0.03) was observed (Table 6). This shift was significantly variable depending on the baseline OSA severity category (*p* < 0.0001). Participants starting with moderate OSA decreased by a mean of 1.4 category level [95% CI, −1.5 to −1.3], while participants with severe OSA decreased by a mean of 2.1 category levels [95% CI, −2.4 to −1.8]. The majority of participants, 91.8% (167/182), were classified as None to Mild severity after MAD treatment.

‘Success’ was achieved in 75.8% (138/182) of participants [95% CI, 68.9–81.8%] according to the criterion of AHI < 10 with MAD combined with at least 50% reduction in AHI from baseline. Additionally, the percent of participants achieving ‘Success’ was similar across OSA severity categories with 76.5% with moderate OSA achieving success compared to 72.7% with severe OSA at baseline (*p* = 0.65).

Tolerance of the prescribed starting position was generally good. One participant required the initial treatment position to be modified from MCP-2 to MCP-4 due to complaints of pain in the temporomandibular joints. Treatment continued without further documented complaints. No treatment discontinuations were recorded during the observation period.

### 3.3. Functional Subjective Outcomes

As shown in Table 4 and Table 5, MAD therapy significantly reduced the snoring loudness with the mean change from baseline equal to −5.4 [95% CI, −5.7 to −5.0] units. None of the baseline characteristics including age, BMI, ESS score and AHI were significant predictors of change in VASS score, except for baseline VASS score. A larger decline in VASS scores from baseline was observed in subjects with higher baseline VASS scores. Participants with moderate OSA had significantly larger decreases from baseline in mean VASS score (Mean change: −5.5 [95% CI, −6.0 to −5.1]) compared to participants with severe OSA at baseline (Mean: −4.5 [95% CI, −5.5 to −3.6]; *p* = 0.049).

Mean ESS improved modestly in the overall cohort (Mean: −1.7 [95% CI, −2.2 to −1.2]). The improvement was similar across baseline OSA severity categories. The additional analysis in the subgroup of 50 participants with baseline ESS ≥ 11 (Table 4) revealed a substantially greater improvement from 14.3 units at baseline to 9.9 units with MAD therapy. Also the mean change from baseline of −4.3 [95% CI, −5.5 to −3.1] showed a statistically significant improvement. The mean change in ESS score was similar across OSA severity categories in this subset. BMI was the only baseline characteristic in addition to baseline ESS, that was determined to be a significant predictor of ESS score change in both the overall cohort and the ‘sleepy’ cohort. In the ‘sleepy’ cohort, the change in ESS score increased by 0.5 units [95% CI, 0.2–0.8] for each additional unit of baseline BMI, thus the reduction in ESS score was more prevalent in participants with lower BMI.

## 4. Discussion

Current guidelines recommend MAD therapy as a first-line treatment for patients with mild OSA and as an alternative for adults with moderate-to-severe OSA who do not tolerate or respond to CPAP therapy [3,9,29]. To our knowledge, this is the first multicenter real-world study evaluating the effectiveness of a specific MAD design in a relatively large cohort of 182 DISE-selected participants with exclusively moderate-to-severe OSA, including 33 participants with severe disease, treated across six general hospitals. Although one large randomized controlled trial involving 220 patients has previously been published [30], real-world multicenter evidence for specific MAD designs remains limited. The present study therefore contributes additional evidence regarding MAD effectiveness in routine clinical practice.

The findings demonstrate substantial improvements in OSA severity following MAD treatment (Table 6). Overall, 41.2% of participants were classified as having no OSA at follow-up, while 91.8% were classified as having either no OSA or mild OSA. No participant remained in the severe OSA category (Figure 3) in the studied cohort. Participants with severe OSA at baseline showed the largest improvements, decreasing by a mean of more than two OSA severity categories. These findings suggest that carefully selected patients with severe OSA may derive considerable benefit from MAD therapy when DISE indicates a favorable response to mandibular advancement. Although severe OSA represented the smaller of the two study subgroups, the observed improvements were substantial and clinically meaningful.

Using the predefined success criterion of AHI < 10 events/h combined with at least a 50% reduction from baseline, 75.8% of participants were successfully treated. This result is consistent with previous reports demonstrating clinically meaningful reductions in AHI following MAD therapy [8,11,31]. Notably, treatment success was observed in both moderate and severe OSA categories, supporting the use of MAD therapy beyond traditionally selected mild OSA populations. Treatment effectiveness also was not limited to respiratory parameters: snoring loudness improved substantially (Table 5), with most participants reaching socially acceptable snoring levels. This observation is clinically relevant because snoring is often a major reason for seeking treatment and may influence long-term acceptance of MAD therapy [32].

A key consideration when interpreting the present findings is that treatment outcomes were assessed using Level-3 HPG, whereas diagnosis was established using Level-1 PSG. This reflects the routine Belgian clinical pathway and reimbursement framework for MAD treatment. Nevertheless, PSG and HPG are not interchangeable methods. Because HPG uses total recording time rather than total sleep time, respiratory event indices may be lower than PSG-derived AHI values. Consequently, treatment effects observed in the present study may appear more favorable than if follow-up PSG had been performed. This limitation should be interpreted within the context of the existing literature: the reported differences between the two study levels are well-characterized and largely attributable to methodological differences in event normalization and sleep-time estimation [33,34]. Differences between both study levels vary also according to device type, scoring methodology, patient characteristics, and disease severity: most studies (ApneaLink Air (ResMed, San Diego, CA, USA), Somnotouch (SOMNOmedics AG, Randersacker, Germany) reported mean differences between PSG-derived AHI and HPG-derived respiratory event indices ranging from approximately 6 to 8 events/h (average of 7 events/h), and if one also includes the Spider SAS (MicroPort CRM, Clamart, France) the adjustment factor is more like 4.8/h [35]. So the magnitude varies according to recording device, scoring methodology, patient characteristics, and disease severity. As an exploratory illustration, applying an upward adjustment of 7 events/h to the observed HPG-derived AHI values the success rate drops to 14.8% and to 35.2% when applying the adjustment of 4.8/h. Applying the Belgian threshold of 15/h instead of 10/h in the success criterion yields an 80.2% success rate for the present results, 31.3% with an adjusted average of 7/h and 51.7% with 4.8/h. These exploratory scenarios illustrate that interpretation of HPG-derived outcomes is highly dependent on the assumptions used to estimate PSG-equivalent values. Because no universally accepted conversion exists, such recalculations should be regarded only as illustrative and not as validated estimates of treatment outcome. Accordingly, the primary analyses reported in this study are based on the observed HPG measurements obtained within the Belgian routine clinical pathway.

The relatively low baseline ESS-scores indicate that excessive daytime sleepiness was not a prominent symptom in most participants. This observation is consistent with previous studies demonstrating that not all patients with OSA report clinically relevant daytime sleepiness [36] and does not necessarily indicate the absence of clinically relevant disease. Nevertheless, participants with elevated baseline ESS scores experienced clinically meaningful improvements following MAD therapy, suggesting that symptomatic benefit may be greatest among patients with pre-existing daytime sleepiness.

Although oxygen-related parameters such as oxygen desaturation index (ODI), oxygen nadir, and time spent below 90% oxygen saturation are clinically relevant, these variables were not consistently available across all participating centers in this retrospective multicenter cohort and were therefore not included in the predefined analyses. Consequently, the present findings primarily reflect changes in respiratory event frequency rather than the full physiological burden of OSA.

Age was associated with treatment outcome, with younger participants demonstrating larger reductions in AHI. This finding is consistent with previous reports identifying younger age as a predictor of favorable MAD response [37,38]. Age-related changes in upper-airway collapsibility, muscle tone, and soft tissue elasticity may contribute to the reduced effectiveness in older patients [38].

BMI showed a modest association with treatment response. Previous studies have reported inconsistent findings regarding the relationship between BMI and MAD effectiveness [8,37,39]. The limited BMI range in the present cohort may partly explain this observation. More broadly, BMI alone may not adequately capture the anatomical and physiological factors influencing treatment response, including upper-airway anatomy and non-anatomical OSA traits [40,41,42].

No sex-related differences in treatment response were observed. Previous studies have reported heterogeneous findings, with some suggesting higher success rates among women and others reporting no significant differences after adjustment for confounding variables [43,44]. Because many MAD studies remain underpowered to evaluate sex-specific treatment effects, larger prospective cohorts are required to further clarify this relationship.

An important characteristic of the present cohort is that all participants underwent DISE prior to treatment initiation. Although DISE has become increasingly standardized, interpretation remains dependent on sedation protocols, scoring systems, and clinical experience [25,45]. Because no mandatory standardized DISE scoring system or formal inter-center calibration was imposed, some variability in patient selection between centers cannot be excluded. This reflects the real-world nature of the study but may limit generalizability. Furthermore, DISE likely enriches the study population for participants with upper-airway collapse patterns that are responsive to mandibular advancement [46]. Consequently, the observed treatment effectiveness should be interpreted as conditional upon DISE-based patient selection and should not be generalized directly to unselected OSA populations [47].

The present study has several important strengths.

- It represents a relatively large (N = 182) multicenter cohort consisting exclusively of DISE-selected participants with moderate-to-severe OSA.
- Identical treatment protocols were applied across six participating hospitals, supporting consistency in patient selection and treatment delivery.
- In addition, participants were allowed to optimize device titration according to symptomatic response prior to objective outcome assessment, thereby reflecting real-world clinical practice.

Several limitations should also be considered. They restrict the generalizability of the present findings, which should be interpreted as descriptive and exploratory, underscoring the need for prospective, controlled studies to confirm these results.

- The study was retrospective and based on routinely collected clinical data, making selection bias unavoidable and limiting causal inference.
- No control group was included, preventing direct comparison with alternative treatments such as CPAP, surgery, or no treatment.
- Follow-up was limited to approximately six months, and longer-term studies are needed to evaluate the durability of treatment effects.
- Because participants were selected following DISE and outcomes were assessed using Level-3 HPG, both patient selection and outcome measurement may have influenced the observed treatment effectiveness. These factors should be considered when interpreting the findings and when comparing the present results with studies using different selection strategies or outcome measures.
- Side effects of MAD are well-documented and have been shown to typically be mild and transient [48] and were not systematically recorded. Accordingly, the present study was not designed to evaluate the safety or tolerability profile of MAD therapy. Routine clinical documentation typically focuses on persistent or clinically significant complaints, and mild or self-limiting symptoms such as jaw discomfort, oral dryness, hypersalivation, or transient occlusal changes may therefore have been underreported [49]. One participant required modification of the initial treatment position because of intolerance, after which treatment continued without further documented complaints. However, the absence of documented adverse events should not be interpreted as evidence that such events did not occur. No treatment discontinuations due to adverse effects were documented during the observation period. Prospective studies with active adverse-event monitoring are needed to better characterize the frequency, duration, and clinical significance of these effects.
- Adherence is an important consideration when interpreting treatment effectiveness. In the present study, device use was assessed by patient self-report rather than objective compliance monitoring. Self-reported adherence may overestimate actual nightly use [27], particularly within healthcare systems where continued reimbursement depends on demonstrating treatment compliance. Nevertheless, self-report remains the most commonly available adherence measure in routine clinical practice and is therefore consistent with the real-world nature of the present study.
- The present study focused on treatment effectiveness as assessed by AHI, OSA severity category, snoring loudness, and daytime sleepiness, which represent the principal outcome measures used within the Belgian reimbursement pathway for MAD therapy. Because oxygen-related metrics were not consistently available in the retrospective clinical records across all participating centers, the present study cannot fully characterize changes in nocturnal hypoxemic burden following MAD therapy.
- Because the study was retrospective and based on routinely collected clinical data from six independent centers, the analysis was intentionally restricted to predefined outcomes that were consistently available across all participating centers. Future prospective studies may further explore the relationship between MAD therapy and additional respiratory parameters.
- Finally, the study was conducted within the Belgian healthcare system, where reimbursement criteria and referral pathways may differ from those in other countries. These differences should be considered when extrapolating the findings to other healthcare settings.

## 5. Conclusions

Despite methodological differences between polysomnography (Level-1 PSG) and polygraphy (Level-3 HPG), this retrospective multicenter study provides real-world data on the outcomes of a patient-specific MAD treatment in 182 DISE-preselected adults with moderate-to-severe obstructive sleep apnea and one MAD design. Because the study was retrospective, lacked a control group, and assessed outcomes using Level-3 HPG, the findings should be interpreted as exploratory effectiveness data and confirmed in prospective controlled studies.

Overall, the present findings within the DISE-selected cohort demonstrate significant improvements in AHI, snoring loudness and daytime sleepiness following MAD treatment. Treatment effectiveness was observed across both moderate and severe OSA categories. Participants with severe OSA experienced substantial improvements following MAD therapy. These findings support further investigation of MAD therapy in appropriately selected patients with severe OSA in prospective controlled studies.

Given the cohort size and multicenter design this study contributes to the limited body of real-world evidence on MAD therapy in moderate-to-severe OSA following DISE-based patient preselection and within an HPG-based follow-up pathway. Prospective controlled studies with longer follow-up are necessary to confirm these findings.

## Figures and Tables

**Table 1:** Descriptive statistics of baseline characteristics of study population, per center as well as pooled (in italics); *p*-values based on ANOVA).

Center	N	Age (Years) Mean ± SD (Median)	BMI (kg/m^2^) Mean ± SD (Median)	AHI (Events/h) Mean ± SD (Median)	VASS Score (1–10) Mean ± SD (Median)	ESS Score (0–24) Mean ± SD (Median)	ESS-Score ≥ 11 (0–24) Mean ± SD (Median)
AZMO	18	59.0 ± 12.7(60.5)	27.7 ± 3.8 (27.5)	25.0 ± 11.2 (20.8)	7.3 ± 2.2 (8.0)	5.2 ± 4.3 (4.5)	14.5 ± 2.1 (14.5)
AZSM	60	46.1 ± 12.1 (46.0)	27.8 ± 3.7 (27.4)	21.9 ± 6.8 (20.8)	7.2 ± 2.0 (7.0)	8.0 ± 5.4 (7.0)	14.3 ± 3.2 (13.5)
AZVK	19	52.8 ± 7.1 (54.0)	27.4 ± 4.4 (26.0)	21.2 ± 9.1 (18.4)	7.3 ± 2.5 (8.0)	5.9 ± 4.5 (5.0)	13.3 ± 1.3 (13.0)
HHAR	16	48.9 ± 10.2 (51.0)	28.4 ± 4.2 (27.8)	23.0 ± 7.8 (20.7)	7.1 ± 2.4 (7.5)	6.8 ± 3.6 (7.0)	12.5 ± 2.1 (12.5)
IMEL	34	48.6 ± 9.9 (50.0)	29.7 ± 4.8(29.0)	26.9 ± 10.1 (24.3)	7.5 ± 2.1 (8.0)	6.4 ± 4.3 (6.0)	14.2 ± 3.8 (12.0)
VITA	35	46.2 ± 10.5 (46.0)	26.3 ± 3.2 (25.8)	28.2 ± 11.7 (26.7)	6.2 ± 3.2 (7.0)	9.6 ± 5.7 (9.0)	14.7 ± 2.4 (14.0)
*Pooled*	*182*	*48.8 ± 11.4 (50.0)*	*27.9 ± 4.1 (27.2)*	*24.4 ± 9.6 (21.5)*	*7.1 ± 2.4 (7.0)*	*7.4 ± 5.1 (7.0)*	*14.3 ± 2.7 (14.0)*
*p*-value		0.0004	0.03	0.01	0.26	0.01	0.88

Abbreviations: AZMO (AZ Monica); AZSM (AZ Sint-Maarten); AZVK (AZ Voorkempen); HHAR (Heilig Hart Ziekenhuis Lier); IMEL (Imelda Ziekenhuis Bonheiden); VITA (VITAZ); N (number of participants); ± (plus/minus); BMI (body mass index); kg/m^2^ (kilogram per square meter); events/h (events per hour sleep); AHI (apnea–hypopnea index); h (hour); VASS score (score on visual analog scale for snoring); ESS score (score on Epworth Sleepiness Scale); ESS score ≥ 11 (score on Epworth Sleepiness Scale in subgroup with ESS ≥ 11 at baseline); ANOVA (analysis of variance).

**Table 2:** Descriptive statistics of baseline characteristics of categorized study population for OSA severity category and sex, per center as well as pooled (in Italics); *p*-values based on Fisher’s exact test).

Center	N	OSA Severity Category		Sex	
		Moderate % (N)	Severe % (N)	Male % (N)	Female % (N)
AZMO	18	77.8% (14)	22.2% (4)	61.1% (11)	38.9% (7)
AZSM	60	91.7% (55)	8.3% (5)	73.3% (44)	26.7% (16)
AZVK	19	89.5% (17)	10.5% (2)	52.6% (10)	47.4% (9)
HHAR	16	81.3% (13)	18.7% (3)	68.7% (11)	31.3% (5)
IMEL	34	76.5% (26)	23.5% (8)	97.1% (33)	2.9% (1)
VITA	35	68.6% (24)	31.4% (11)	80.0% (28)	20.0% (7)
*Pooled*	*182*	*81.9% (149)*	*18.1% (33)*	*75.3% (137)*	*24.7% (45)*
*p*-value		0.07		0.002	

Abbreviations: AZMO (AZ Monica); AZSM (AZ Sint-Maarten); AZVK (AZ Voorkempen); HHAR (Heilig Hart Ziekenhuis Lier); IMEL (Imelda Ziekenhuis Bonheiden); VITA (VITAZ); OSA (obstructive sleep apnea); % (percentage of participants); N (number of participants).

**Table 3:** Analysis of poolability across centers for primary outcome, AHI change from baseline (unadjusted *p*-value includes only center as explanatory variable in the model; adjusted *p*-value includes center, age, baseline AHI and baseline BMI as explanatory variables in the model; pooled data in italics).

Center	N	Mean Age Baseline (Years)	Mean BMI Baseline (kg/m^2^)	Mean AHI Baseline (Events/h)	AHI + MAD Change from Baseline (Events/h)			
					Unadjusted Mean ± SE	Unadjusted 95% CI	Adjusted Mean ± SE	Adjusted 95% CI
AZMO	18	59.0	27.7	25.0	−17.7 ± 2.7	−23.4 to −12.1	−18.8 ± 1.1	−21.0 to −16.6
AZSM	60	46.1	27.8	21.9	−14.7 ± 1.1	−16.9 to −12.4	−16.4 ± 0.6	−17.5 to −15.2
AZVK	19	52.8	27.4	21.2	−12.2 ± 2.1	−16.7 to −7.8	−15.7 ± 1.1	−17.8 to −13.6
HHAR	16	48.9	28.4	22.9	−17.3 ± 2.0	−21.5 to −13.0	−18.7 ± 1.1	−20.9 to −16.4
IMEL	34	48.6	29.7	26.9	−20.0 ± 1.7	−23.6 to −16.5	−18.2 ± 0.8	−19.8 to −16.7
VITA	35	46.2	26.3	28.2	−21.7 ± 1.8	−25.3 to −18.1	−17.5 ± 0.8	−19.0 to −15.9
*Pooled*	*182*	*48.8*	*27.9*	*24.4*	*−17.3 ± 0.7*	*−18.8 to −15.8*	*−17.3 ± 0.3*	*−18.0 to −16.6*
*p*-value testing poolability					0.002		0.100	

Abbreviations: AZMO (AZ Monica); AZSM (AZ Sint-Maarten); AZVK (AZ Voorkempen); HHAR (Heilig Hart Ziekenhuis Lier); IMEL (Imelda Ziekenhuis Bonheiden); VITA (VITAZ); N (number of observations); events/h (events per hour sleep); ± (plus/minus); AHI (apnea–hypopnea index); AHI + MAD (apnea–hypopnea index with mandibular advancement device); SE (standard error); 95% CI (confidence interval for the mean); N (number of participants).

**Table 4:** Overall effect of mandibular advancement device treatment on primary and secondary outcome measures (change from baseline for AHI presented as change and %change; mean change from baseline, 95% CI’s and *p*-values based on ANCOVA adjusting for baseline AHI).

Outcome Measure	N	Baseline	Follow-Up + MAD	Change from BL	Adjusted 95% CI	*p*-Value
		Mean ± SD (Median)	Mean ± SD (Median)	Mean ± SE		
AHI (events/h)	182	24.4 ± 9.6 (21.5)	7.1 ± 5.1 (5.9)	−17.3 ± 0.4	−18.0 to −16.6	<0.0001
AHI (%change)	182	24.4 ± 9.6 (21.5)	7.1 ± 5.1 (5.9)	−68.6% ± 1.7%	−72.0% to −65.2%	<0.0001
VASS score (1–10)	182	7.1 ± 2.4 (7.0)	1.7 ± 1.6 (1.0)	−5.4 ± 0.1	−5.7 to −5.0	<0.0001
ESS score (0–24)	182	7.4 ± 5.1 (7.0)	5.7 ± 4.3 (5.0)	−1.7 ± 0.2	−2.2 to −1.2	<0.0001
ESS score—“sleepy” subset (ESS BL between 11 and 24)	50	14.3 ± 2.7 (14.0)	9.9 ± 4.5 (9.5)	−4.3 ± 0.6	−5.5 to −3.1	<0.0001

Abbreviations: N (number of participants); ± (plus/minus); SD (standard deviation); SE (standard error); 95% CI (95% confidence interval for the mean change or mean percent change); AHI (apnea–hypopnea index); events/h (events per hour sleep); +MAD (outcome with mandibular advancement device); %change (percent change in AHI from baseline); VASS score (score on visual analog scale for snoring); ESS score (score on Epworth Sleepiness Scale); BL (baseline); ANCOVA (analysis of covariance).

**Table 5:** Change in outcome measures by baseline OSA classification (statistics presented are mean ± standard deviation and as median; 95% CI. *p*-value based on Student’s *t*-test).

Outcome Measure	Baseline OSA Severity Classification				*p*-Value
	Moderate OSA		Severe OSA		
	N	Mean ± SD Median [95% CI]	N	Mean ± SD Median [95% CI]	
AHI + MAD (absolute change from BL)	149	−14.1 ± 6.3 −14.2 [−15.1 to −13.1]	33	−31.8 ± 10.9 −31.8 [−35.6 to −27.9]	<0.0001
AHI + MAD (percentage change from BL)	149	−66.6% ± 24.7% −72.7% [−70.6% to −62.6%]	33	−77.9% ± 17.7% −83.4% [−84.2% to −71.7%]	0.003
OSA severity category + MAD (change from BL expressed as the number of categories)	149	−1.4 ± 0.6 −1.0 [−1.5 to −1.3]	33	−2.1 ± 0.8 −2.0 [−2.4 to −1.8]	<0.0001
VASS score + MAD (absolute change from BL)	149	−5.5 ± 2.7 −6.0 [−6.0 to −5.1]	33	−4.5 ± 2.7 −4.0 [−5.5 to −3.6]	0.049
ESS score + MAD overall (absolute change from BL)	149	−1.7 ± 3.3 0 [−2.2 to −1.2]	33	−1.8 ± 3.3 0 [−3.0 to −0.7]	0.85
ESS score + MAD for “sleepy” cohort (absolute change from BL)	41	−4.3 ± 3.9 −6.0 [−5.5 to −3.1]	9	−4.3 ± 5.2 −6.0 [−8.3 to −0.3]	0.99

Abbreviations: OSA (obstructive sleep apnea); N (number of participants); ± (plus/minus); SD (standard deviation); 95% CI (95% confidence interval for the median presented for pooled results); AHI (apnea–hypopnea index); MAD (mandibular advancement device); AHI + MAD (apnea–hypopnea index with mandibular advancement device); BL (baseline); VASS score + MAD (score on visual analog scale for snoring with mandibular advancement device); ESS score + MAD (score on Epworth Sleepiness Scale with mandibular advancement device).

**Table 6:** Effect of mandibular advancement device treatment on OSA severity category (*p*-value based on a trend test).

Baseline OSA Severity Category	OSA Severity Category After MAD Treatment % (N)			*p*-Value
	No OSA	Mild OSA	Moderate OSA	
Moderate (N = 149)	42.3% (63)	53.0% (79)	4.7% (7)	0.03
Severe (N = 33)	36.4% (12)	39.4% (13)	24.2% (8)	

Abbreviations: OSA (Obstructive Sleep Apnea); MAD (mandibular advancement device); % (percent of participants); N (number of participants).

## Data Availability

The datasets generated and analyzed during the current study are not publicly available due to Belgian privacy and hospital regulations, but de-identified data may be made available upon reasonable request to the corresponding author (marc.braem@uantwerpen.be) and with permission from the participating hospitals.

## Abbreviations

The following abbreviations are used in this manuscript:

AASM	American Academy of Sleep Medicine
AHI	apnea–hypopnea index
ANCOVA	analysis of covariance
ANOVA	analysis of variance
AZMO	Algemeen Ziekenhuis Monica Antwerpen
AZSM	Algemeen Ziekenhuis Sint-Maarten, Mechelen
AZVK	Algemeen Ziekenhuis Voorkempen, Malle
BMI	body mass index
CI	confidence interval
CPAP	continuous positive airway pressure
DISE	drug-induced sleep endoscopy
ENT	ear, nose and throat
ESS	Epworth Sleepiness Scale
ESS score	score on Epworth Sleepiness Scale
events/h	events per hour sleep
F	female
HHAR	Heilig Hart Ziekenhuis, Lier
HPG	home polygraphy
IMEL	Imelda Ziekenhuis, Bonheiden
L	lower
M	male
MAD	mandibular advancement device
MCP	maximal comfortable protrusion
N	number of participants
OSA	obstructive sleep apnea
PG	polygraphy
PSG	polysomnography
RACEMADT	Real-World Assessment of Clinical Evidence for Mandibular Advancement Treatment of Obstructive Sleep Apnea
RCT	randomized clinical trial
SD	standard deviation
SE	standard error
U	upper
VASS score	score on visual analog scale for snoring loudness
VITA	Algemeen Ziekenhuis Nikolaas and Algemeen Ziekenhuis Lokeren fusion, ‘VITAZ’, Sint-Niklaas

## Appendix A

### Appendix A.1

The details of the polysomnography registrations are as follows: at AZMO: SOMNOscreen, SOMNOmedics AG, Randersacker, Germany with software Domino 2.9.0; at AZSM: SOMNO HD/eco Type NGD050, SOMNOmedics AG, Randersacker, Germany with software Domino 3.1.0.0; at AZVK: Dream, Medatec Medical Data Technology, Braine-le-Château, Belgium with Brainnet 5.14.0.0; at HHAR: SOMNOscreen Plus, SOMNOmedics AG, Randersacker, Germany with software Domino; at VITA: Brainnet 5.14.0.0, Medatec Medical Data Technology, Braine-le-Château, Belgium with Brainnet for Windows 5.24.0.0). Recordings included electroencephalogram channels from frontal (F4–M1), central (C4–M1), and occipital (O2–M1) derivations; electro-oculogram channels from the left and right eyes (E1–M2 and E2–M2); submental (chin) and bilateral tibialis electromyogram channels; and a single-lead electrocardiogram; airflow via nasal pressure transducer and thermistor, thoracoabdominal inductance plethysmography, pulse oximetry, and body position sensor; video and sound monitoring were used.

### Appendix A.2

The details of the polygraphy registrations are as follows: at AZMO: SOMNOtouch Resp, SOMNOmedics AG, Randersacker, Germany with software Domino-light 1.5; at AZSM: SOMNOtouch: RES, SOMNOmedics AG, Randersacker, Germany with software Domino 3.1.0.0; at AZVK; Dreamscan, Medatec Medical Data Technology, Braine-le-Château, Belgium with Brainnet Software; at IMEL: SOMNOtouch Resp Eco, SOMNOmedics AG, Randersacker, Germany with software Domino-light 1.5; at HHAR: SOMNOtouch Resp Eco, SOMNOmedics AG, Randersacker, Germany with software Domino-light; at VITA: MediByte (Braebon Medical Corporation, Ottawa, ON, Canada) with software MediByte vs. 8.0) within five months after treatment initiation and during a six month observation period. Recorded channels included nasal airflow via pressure transducer and thermistor, thoracic and abdominal respiratory effort belts, pulse oximetry for oxygen saturation and heart rate, and a body position sensor. Snoring was recorded using an integrated microphone. No electroencephalogram, electrooculogram, or electromyogram channels were recorded, not allowing sleep staging.

## Appendix B

Poolability across centers was tested based on a univariate approach considering center as the only explanatory variable (unadjusted) and a multivariate approach additionally adjusting for baseline characteristics (adjusted). Figure A1 displays the unadjusted and adjusted results for each center. This figure shows how adjusting for unbalanced baseline characteristics tightens the 95% confidence intervals and makes them more comparable between centers. This is further illustrated in Figure A2 that shows how close together the mean change from baseline is based on the adjusted results.
